# Protective and therapeutic effects of *Lactobacillus brevis* PQ214320 and *Bacillus subtilis* PQ198038 Against experimental *Trichinella* Infection

**DOI:** 10.1371/journal.pntd.0013331

**Published:** 2025-08-11

**Authors:** Eman E. El Shanawany, Rania Abdel-Razik, Amany Ebrahim Nofal, Rabab S. Zalat, Faten Abouelmagd

**Affiliations:** 1 Parasitology and Animal Diseases Department, Veterinary Research Institute, National Research Centre, Dokki, Giza, Egypt; 2 Chemistry of Natural and Microbial Products Department, Pharmaceutical and Drug Industries Research Institute, National Research Centre, Dokki, Giza, Egypt; 3 Histology and histochemistry, Zoology Department, Faculty of Science, Menoufia University, Menoufia, Egypt; 4 Department of Parasitology, Theodor Bilharz Research Institute, Giza, Egypt; 5 Department of Medical Parasitology, Faculty of Medicine, Sohag University, Sohag, Egypt; Salk Institute for Biological Studies, UNITED STATES OF AMERICA

## Abstract

One of the most significant lactic acid bacteria genera is *Lactobacillus*, which is known to generate compounds such as bacteriocins that can stop the growth of harmful bacteria. The current study investigated the protective and therapeutic effects of two novel probiotic strains, *Lactobacillus brevis* PQ214320, and *Bacillus subtilis* PQ198038, on parasitological, histopathological, and immunological responses in mice infected with *Trichinella spiralis*. A total of 120 mice were divided into six groups, including a positive control group (C) which was infected with *Trichinella* infection and not treated, mice treated orally with albendazole (ALB) at a dose of 5 mg/kg bw for 3 days after infection, and mice treated with probiotics (10^9^ Colony-Forming Unit (CFU)/mL/animal, in 100 µL of Ringer’s solution) either pre- and post-infection with *L. brevis* PQ214320 (LP) or *B. subtilis* PQ198038 (BSP), or only post-infection with *L. brevis* PQ214320 (L) and *B. subtilis* PQ198038 (BS). Infection was induced by oral inoculation of 400 *T. spiralis* larvae. Parasite burden and, histopathological, and immune responses were assessed at 5 and 19 days post-infection. The results showed that the LP group had significantly reduced adult worm and muscle larval counts compared with the positive control group. In contrast, BSP reduced the parasite burden, but to a lesser extent. The immune response was characterized by elevated levels of IL 12 and IFN-γ in the LP group at 5 days -post-infection (dpi), indicating a strong Th1 response, which declined but remained significantly higher than in the control infected group at 19 dpi. Serum IgG responses were higher in the LP group at 19 dpi, suggesting that a more robust adaptive immune response was triggered by *L. brevis*. Pre- and post-treatment with *B. subtilis* PQ198038 and *L. brevis* PQ214320 significantly improved the histopathological abnormalities and collagen deposition in the small intestinal and diaphragm muscular tissues caused *Trichinella* infection and restored claudin 1 content in the same tissues. These findings suggest that *L. brevis* PQ214320 offers a stronger protective effect against *T. spiralis* infection, potentially through enhanced immune modulation and parasite reduction, whereas *B. subtilis* PQ198038 provides beneficial but less potent responses. This study highlights the potential of novel probiotics strains as adjunct protective agents and therapies against *T. spiralis* infection.

## Author summary

*Trichinella spiralis* is a parasitic disease that affects both humans and animals. Probiotics, beneficial bacteria that support gut health and immune function, have emerged as promising alternative or complementary therapies. In this study, we investigated the protective and therapeutic effects of two novel probiotic strains, *Lactobacillus brevis* PQ214320 and *Bacillus subtilis* PQ198038, in mice infected with *T. spiralis*. Mice receiving *L. brevis* PQ214320 before and after infection showed the most significant reductions in parasite burden and improvements in immune responses, particularly through enhanced Th1 immunity (elevated IL 12 and IFN-γ) and stronger adaptive responses (higher IgG levels). Histopathological observations revealed that both probiotics alleviated intestinal and muscular damage caused by the parasite and helped restore the integrity of tissue barriers. However, *L. brevis* PQ214320 demonstrated protective effects compared to *B. subtilis* PQ198038. Our findings suggest that probiotics, especially *L. brevis* PQ214320, may serve as effective adjunct therapies for *Trichinella* infection by modulating the immune response and reducing parasite load. These results provide new insights into probiotic-based interventions for parasitic infections, offering potential benefits for both human and veterinary medicine.

## 1. Introduction

*Trichinella spiralis*, a parasitic nematode, is the etiological agent of *Trichinella* infection and is characterized by an extremely wide host range and worldwide distribution [1,2]. In the global ranking of food-borne parasites, *T. spiralis* was listed among the top ten [3]. From both medical and veterinary perspectives, humans, swine, and horses are considered the most significant hosts of this parasite [4]. Human infection occurs upon ingestion of undercooked or raw meat contaminated with the infective larvae of the parasite [5,6]. The infection primarily affects the gastrointestinal tract, followed by systemic dissemination of the larvae to the muscles, resulting in symptoms such as myalgia, fever, and, in severe cases, cardio pulmonary complications [6]. *T. spiralis* is the most pathogenic and widespread species causing *Trichinella* infection in humans [7,8]. Throughout its life cycle, *T. spiralis* induces inflammation in both the intestines by the adult worm and muscles by the larvae [9].

Therapeutic approaches to *Trichinella* infection can generally primarily involve the use of anthelmintics, specifically albendazole and mebendazole [10,11]. Although anthelmintics such as albendazole and mebendazole are commonly used in the early intestinal phase of *Trichinella* infection, their effectiveness becomes limited once the larvae migrate to an encyst within the muscles. At this stage, the disease is primarily managed by addressing the intense inflammatory response triggered by the encysted larvae [12]. Therefore, corticosteroids are considered the mainstay of treatment in the muscular phase to alleviate inflammation and associated symptoms. Moreover, the efficacy of these benzimidazole derivatives is limited by several factors: 1) weak activity against encapsulated larvae, 2) poor water solubility, 3) rising anthelmintic resistance, and 4) contraindications in children and during pregnancy [13].

Recent limitations in traditional therapeutic strategies have led to an increased interest in the exploration of novel treatment options. In particular, alternative therapies have attracted considerable attention. Probiotic bacteria have been highlighted as potential therapeutic agents [14], while natural proteins have been explored for their effectiveness [15].

Helminth infections cause significant changes in the composition of the gut microbiota [16,17]. The products and metabolites produced by the gut microbiota also have a considerable impact on the survival and physiology of many parasites, thereby influencing the overall outcome of parasitic infections. This suggests that probiotic bacteria can effectively reduce the pathogenicity of numerous parasites, through various mechanisms [18,19]. The key mechanisms of probiotic action include strengthening the gut epithelial barrier, enhancing adhesion to the intestinal mucosa, inhibiting pathogen attachment, competitively eliminating pathogens, producing antimicrobial compounds, and modulating the immune system [20].

Exogenous live microorganisms known as probiotics, when given into the digestive tract improve the gut health of the host [21]. Most probiotics currently in use are comprise lactic acid bacteria, especially *Lactobacilli*, however, they also contain enterococci, bifidobacteria, and streptococci [22]. *Lactobacilli* colonize several parts of the human body, most notably the digestive tract including the oral cavity, and female vagina. Humans and *Lactobacilli* have a mutualistic connection in which *Lactobacillus* species provide host digestion of specific food substrates, as well as defense against infections, in exchange for housing and nourishment [23,24]. *Lactobacilli* have been approved as safe by the U.S. Food and Drug Administration and the European Food Safety Authority, making *Lactobacilli* highly studied probiotic candidates [25].

The mechanisms by which probiotics exert their antiparasitic effects are complex and multifaceted. One potential mechanism involves the modulation of the host immune response [26–28]. Probiotics can stimulate the production of cytokines and other immune mediators that can directly target the parasite or create an unfavorable environment for its survival [29]. The antiparasitic properties of probiotic bacteria have garnered increasing attention over the years [14]. Probiotics reduce the parasite load and associated pathological changes in experimental *Trichinella* infection by enhancing both local and systemic immune responses [14,30,31]. In addition to their use in treatment, it plays an integral protective role against *Trichinella* and when compared to traditional attenuated vaccines, probiotics-based recombinant vaccines are regarded as an excellent option for veterinary use [32].

Previous data emphasize the pressing need to identify microbial strains with well-defined characteristics that can be integrated into human health interventions. These strains should be effective in preventing and treating helminth infections, while also shedding light on the mechanisms through which probiotics exert their antiparasitic effects. Therefore, the present study aimed to cultivate and characterize two strains, *Lactobacillus brevis* PQ214320*,* and *Bacillus subtilis* PQ198038, and evaluate their protective and therapeutic effects against *Trichinella* infection. The efficacy of these strains will be assessed by measuring immunological markers such as IgG, IL 12, IFN-γ, and IL 17, along with analyzing histopathological changes, and immunohistochemical expression of Claudin-1 in the small intestine and diaphragm muscle. Parasitological evaluations were conducted by counting the number of adult worms and larvae during the intestinal and muscular phases of the infection.

## 2. Materials and methods

### 2.1. Ethics Statement

The experimental animals were maintained in compliance with ethical rules according to the Guidelines for Care and Use of the Institutional Animal Care and Use Committee (IACUC) Faculty of Science Menoufia University, Egypt (No: MUFS/F/HI/2/23).

### 2.2. Experimental study design

**Fig 1:**
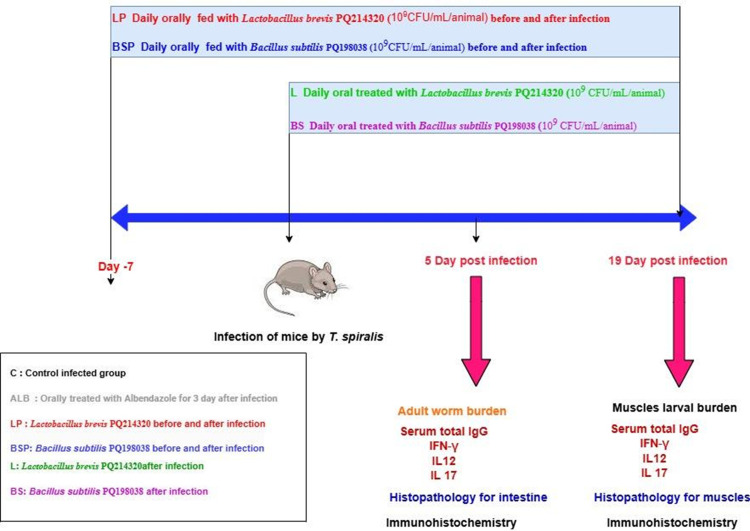
Infographic to show timings, sample collection, and processing (https://app.diagrams.net/).

One hundred and twenty Swiss albino male mice aged 8–10 weeks were divided equally into six groups (Fig 1). Group 1 (C) served as the positive control and was infected with *T. spiralis* without treatment. Group 2 (ALB) consisted of mice administered albendazole at a dose of 5 mg/kg body weight for 3 days after infection. Group 3 (LP) included mice that were daily orally administered with *L. brevis* at a dose of 10^9^ Colony-Forming Unit (CFU)/mL/animal, in 100 µL of Ringer’s solution, starting 7 days before infection and continuing until the end of the experiment. Group 4 (BSP) comprised mice that were daily orally administered *B. subtilis* at a dose of 10^9^ CFU/mL/animal, in 100 µL of Ringer’s solution, starting 7 days before infection and continuing until the end of the experiment. Groups 5 (L) and 6 (BS) involved mice treated with daily oral administration of *L. brevis* and *B. subtilis*, respectively, at a dose of 10^9^ CFU/mL/animal, in 100 µL of Ringer’s solution until the end of the experiment. Infection was induced by oral inoculation of mice with 400 *T. spiralis* muscle larvae [33]. Blood and small intestine samples were collected from ten mice in each group on day 5 post-infection. The remaining mice in each group were sacrificed on day 19 post-infection to collect blood and muscle samples. Serum samples were isolated by centrifugation at 4000 × *g* for 5 min, and the sera were collected and stored at −20 °C until further use.

### 2.3. Probiotic growth, and identification

#### 2.3.1. Probiotic and growth medium.

*L. brevis* and *B. subtilis* utilized in the current study were obtained from the Chemistry of Natural and Microbial Products Department National Research Centre and were identified by 16s rRNA and biochemical test. Microorganisms were maintained for growth on De Man, Rogosa, and Sharpe (MRS) broth medium for 24 h at 37 °C, pH 6.2, and nutrient broth respectively. Cultures were kept in a refrigerator at 4 °C for preservation.

#### 2.3.2. *B. subtilis* and *L. brevis* counting.

A large number of microorganisms were observed in the turbid broth tubes. However, it was not possible to estimate the bacterial cell count on agar plates, as the colonies were too numerous to count. Thus, before plating, we must dilute the original sample to get a countable plate (30–300 bacteria). The number of colonies on a plate provides the CFU or colony-forming units. When the CFU was divided by the plated volume, the result was expressed in CFU/mL.

**Inoculation process:** By sterile pipette 1 ml of the inoculum (sample) was placed inside a sterile petri dish (diameter 90 mm), and 20 mL of plate count medium was spread on the inoculated plates and mixed well with the inoculum sample. When inoculated plates were completely solidified, they were inverted and placed in the incubator at 35 °C ± 2 °C, taking care that the dishes were separated from one another and the walls and the top of the incubator. The duration of incubation for this bacterial strain was 24–48 hours [34].

#### 2.3.2. Safety of *B.subtilis* and *L. brevis.*

**Antibiotic susceptibility of strains:** To be utilized in humans, probiotics need to have a favorable safety profile. The strains’ resistance to a wide range of drugs and lack of pathogenicity are especially important. We investigated the susceptibility to six antibiotic discs using the agar diffusion technique: Penicillin (P10) Erythromycin (E15), Amoxicillin (AMC-30), Azithromycin (AZM 15), and Ciprofloxacin (Cip). The results were categorized as sensitive, moderate, and resistant after the inhibitory zone diameters were measured [35]

**Antimicrobial activity assay:** Agar well diffusion assay was utilized to assess antibacterial activity [36]. At the proper temperatures for the growth of the indicator strains, plates were incubated for 48 hours inhibition zones were read following 48-hour incubation [37,38].

### 2.4. Parasitological evaluations

#### 2.4.1. Preparation of *T. spiralis* larvae for infection.

*T. spiralis* strain was maintained and species were identified at the Parasitology Department, Theodor Bilharz Research Institute, Egypt by serial passage in Swiss strain albino mice as described by Wassom et al. [39]. Muscle tissue from infected mice was collected and ground using a meat grinder. The ground muscle was then digested in a solution containing 1% HCl and 1% pepsin, as described by Martínez-Gómez et al. [40]. This mixture was incubated in a water bath at 37 °C for 2 h. Following incubation, the solution was filtered through sieves with mesh sizes of 50, 100, and 200, and washed with tap water. The larvae were collected and counted using a nematode-counting chamber under a microscope.

#### 2.4.2. Parasite Burden.

To assess the intestinal phase of *Trichinella* infection, worms were isolated from the small intestine 5 dpi. Small segments of the intestine (5–10 cm each) were incubated on sieves placed in conical glasses containing 0.9% saline solution at 37 °C overnight. The next day, the worms in the sediment were counted using a microscope.

The evaluation of the muscular phase of *Trichinella* infection was based on the number of larvae isolated from the muscles at 19 dpi. Following the method described by Martínez-Gómez et al. [40], the tissue mass of each mouse was ground and digested with 1% HCl and 1% pepsin. The larvae from the resulting sediment were counted under a microscope at 40 × magnification. The results were presented as the number of larvae recovered per mouse.

#### 2.4.3. Larval antigen preparation.

The antigen was prepared from *T. spiralis* larvae according to the method described by Martínez-Gómez et al. [35]. Briefly, *T. spiralis* muscle larvae were suspended in 10 mM Tris-HCl containing protease inhibitors and homogenized at 4 °C using a tissue homogenizer. The resulting suspension was centrifuged at 14,000 × g at 4 °C for 30 minutes using a cooling centrifuge. The protein content of the supernatant collected antigen was determined according to Lowry et al. [41]. Then the collected antigen was aliquoted and stored at −20 °C until use.

### 2.5. Enzyme-Linked Immunosorbent Assay for Immunological Assessment

#### 2.5.1. *T. spiralis* specific IgG Assay.

An indirect Enzyme-Linked Immunosorbent Assay (ELISA) was used to determine the presence of *T. spiralis*-specific IgG at 5 and 19 dpi. ELISA was performed according to the method of Connick et al. [42]. Checkerboard titration was performed to determine the optimal concentrations of the antigen, conjugate, and serum dilutions. The ELISA plate was coated with *T. spiralis* larvae antigen at a dilution of 30 μg/ml and then incubated at 4 °C for 24h. The plate then was washed three times with PBS-Tween 20, 100 microliters of blocking buffer was added to each well, and the plate was for 1 h at room temperature. The plate was washed three times with PBS-Tween 20, then added to each well 100 μl of tested serum diluted (1:100) in PBS-Tween. The plate was then incubated for 1.5 h at 37 °C and washed three times with PBS-Tween 20. Peroxidase-labeled anti-mouse IgG horse radish peroxidase (Prod. No. A4416 Sigma, USA) was diluted 1: 1000, and 100 microliters were added to each well, then incubated the plate for 1 h at 37°C. After washing the plate, 100 μl of orthophenylenediamine was added to each well and incubated the plate in the dark for 20–30 min. The absorbance was read at 405 nm on an automatic micro ELISA reader ELx 800 (BIOTEK instrument, INC, Germany). The optical density (OD) cut-off value was determined using the method described by Hegazi et al. [43]. The cut-off value was subtracted from all ODs.

#### 2.5.2. Assessment of IFN-γ, IL 12, and IL 17 in sera.

The levels of IL 12, IL 17, and IFN-γ in mice were assessed at 5 and 19 dpi using commercially available sandwich ELISA kits for each cytokine (Bioneovan Co., Ltd., Beijing, China) and performed according to the protocol provided by the manufacturer.

### 2.6. Histological and histochemical examinations

Small intestine specimens were collected at 5 dpi, and diaphragm muscles were collected at 19 dpi, and were discredited from all mice groups and fixed in 10% neutral buffered formalin (24 h) for paraffin block preparation [44]. Deparaffinized sections (5 µm) were stained with hematoxylin and Eosin (H&E) for histopathological examination, and Picrosirius red (SR) was used to evaluate the organization of collagen fibers in tissue sections [45].

### 2.7. Immunohistochemistry examinations (Claudin-1 expression)

Immunohistochemical reaction was performed using the avidin-biotin-peroxidase complex method following the manufacturer’s instructions. Paraffin small intestinal and diaphragm muscle sections (4 µm) of all mice groups on charged slices were reacted specifically with Claudin-1 rabbit polyclonal antibodies (Cab15098; dilution, 1:200; Abcam, Cambridge, MA, USA) [46] according to the manufacturer’s instructions.

Histological, histochemical, and immunohistochemical sections were photographed with an Olympus BX 41 microscope (Tokyo, Japan). The positive area percentage (%) of red color for the collagen fiber network, and brown immune reactions for Claudin-1 have been quantified by suitable image editing software (Image J, NIH, Bethesda, MD, USA), from 10 random fields per slides (5 slices for each group), and the obtained data were presented as mean ± standard deviation in graphic charts [47].

### 2.8. Statistical data analysis

Data were expressed as mean value ± standard deviation (SD). GraphPad Prism Software (version 6; GraphPad Software, Inc, La Jolla, CA, USA) were used to compute statistics between the groups. Result were considered significant at *P* < 0.05, *P <* 0.001, and *P* < 0.0001 by using one-way ANOVA, post hoc Tukey’s test for multiple comparisons was used. The normality of data distribution was assessed using the Shapiro-Wilk test. The results demonstrated that all groups followed a normal distribution (*P >* 0.05 for all groups). Therefore, parametric statistical analyses, including one-way ANOVA, were appropriately applied.

## 3. Results

### 3.1. Antibiotic susceptibility of strains

Antibiotic susceptibility tests were performed using the agar diffusion method, as shown in Table 1, *B. subtilis* PQ198038 demonstrated resistance to amoxicillin and penicillin, while *L. brevis* PQ214320 was susceptible to all six antibiotics tested: ciprofloxacin (Cip), Azithromycin (AZM 15), Amoxicillin (AMC-30), Vancomycin (VA 30), Penicillin (P10), and Erythromycin (E15).

**Table 1:** Susceptibility of isolated strains to antibiotics.

Isolated strain	Cip	AZM 15	AMC-30	VA 30	P10	E 15
*B. subtilis PQ198038*	Susceptible	Susceptible	Resistant	Susceptible	Resistant	Susceptible
*L. brevis PQ214320*	Susceptible	Susceptible	Susceptible	Susceptible	Susceptible	Susceptible

Note: Ciprofloxacin (Cip), Azithromycin (AZM 15), Amoxicillin (AMC-30), Vancomycin (VA 30), Penicillin (P10), and Erythromycin (E15).

### 3.2. Antimicrobial activity against pathogens

The antimicrobial effects of *B. subtilis* PQ198038 and *L. brevis* PQ214320 showen in Table 2. Both strains inhibited the growth of *Staphylococcus aureus*, *Escherichia coli*, and *Pseudomonas aeruginosa* with inhibition zones ranging from 12 to 23 mm. Neither strain demonstrated activity against *Klebsiella pneumoniae* (Table 2).

**Table 2:** Antimicrobial activity of isolated strains against pathogenic indicators.

Pathogen	Inhibition Zone (mm) –*B. subtilis PQ198038*	Inhibition Zone (mm) – *L. brevis PQ214320*
*Escherichia coli*	12	12
*Staphylococcus aureus*	23	20
*Pseudomonas aeruginosa*	21	19
*Klebsiella pneumoniae*	0	0

### 3.3. Parasitological assessment

#### 3.3.1. Parasite burden: numbers of adults and muscle larvae.

**Fig 2:**
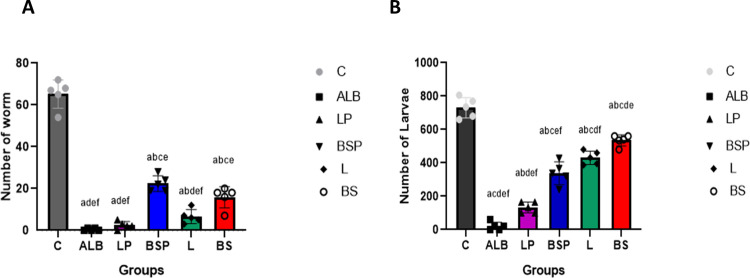
(A) *T. spiralis* adult worms’ numbers in the small intestine of mice (n = 5) treated with probiotics. **(B)** Numbers of muscle larvae isolated from mice (n = 5) with probiotic treatment and *T. spiralis* infection. The significant (*P < *0.01) differences are (A) compared to the C group, (B) against the ALB group, (C) compared with the LP group, (d) compared with the BSP group, (E) compared with L group, and (F) compared with BS group. One-way ANOVA, post hoc Tukey’s test for multiple comparisons was used.

Both *L. brevis* PQ214320 and *B. subtilis* PQ198038 have the potential to reduce the counts of *T. spiralis* adults and larvae but with varying levels of success. Notably, *L. brevis* PQ214320 appeared to be more effective than *B. subtilis* PQ198038 at reducing both adult worms and muscle larvae. In the group treated with *L. brevis* (LP group) both before and after infection, the mean adult worm count was significantly lower post-challenge (2.5 ± 1.8) than in the positive control group (65.25 ± 6.7 worms) (Fig 2A). The larval count also felled considerably (Fig 2B), with 132.5 ± 32.5 larvae per gram in the LP group in comparison to 730.3 ± 60.1 larvae per gram in the positive infected control group, showing that *L. brevis* treatment effectively lowers infection levels. By contrast, *B. subtilis* (BSP group) administered in the same manner (pre- and post-infection) reduced adult worms to 22.5 ± 4.36 and larvae to 338.5 ± 79.1 per gram, while still a significant decrease compared to the positive control (*P <* 0.0001), wasn’t as effective as *L. brevis*. This suggests that *L. brevis* exerts a strong protective effect against *T. spiralis*. The group administrated *L. brevis* only post-infection in the L group resulted in a mean larval count of 430.5 ± 40.4 per gram which was significantly higher than the LP group *P < *0.0001. However, mean worm counts did not differ significantly between the L and LP groups. Similarly, *B. subtilis* given post-infection had a mean larval count of 535.0 ± 32.7 per gram which was significantly higher than the BSP group*.*

### 3.4. Immunological assessment

#### 3.4.1. ELISA determination of cytokine responses.

**Fig 3:**
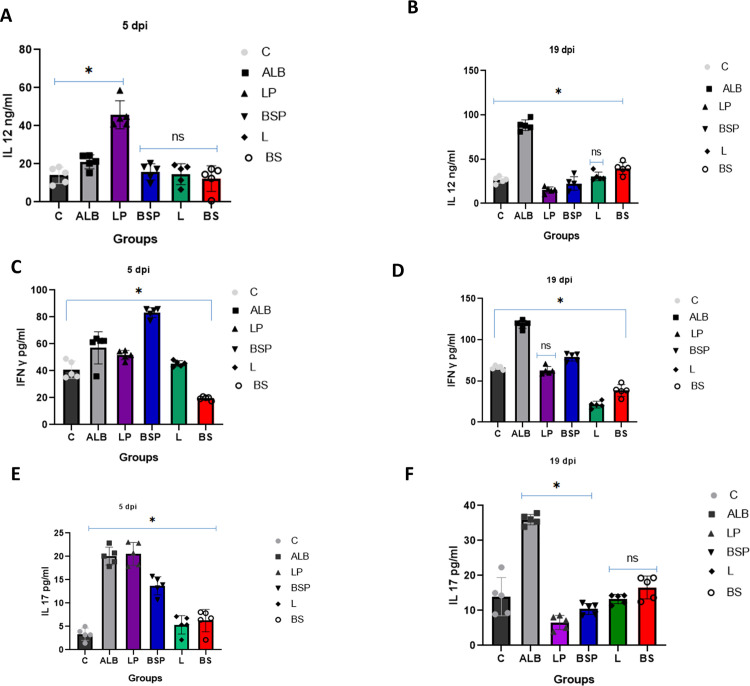
Levels of IL 12(a, b), IFN-γ (c,d), and IL 17 (e,f) from different groups of mice. The data are shown as the mean ± SD of 5 mice per group. The cytokines were quantified using an ELISA. One-way ANOVA, post hoc Tukey’s test for multiple comparisons was used. * Indicate significance difference (*P < *0.05) in comparison with control infected non-treated group **(C)**. (ns) indicate non-significant differences in comparison with the C group.

At 5 dpi, the LP group exhibited the highest IL 12 levels (42.189 ± 2.264), indicating a strong early immune response. The ALB and LP groups also showed significant (**P* *< 0.05) elevated IL-12 levels compared to the positive control. The L and BS groups had the lowest (11.797 ± 5.39 and 10.163 ± 8.56 respectively) IL 12 levels among the treated groups. At 19 dpi, the ALB group showed significantly (**P* *< 0.0001) higher IL-12 levels than the other groups. The positive control group maintained moderate IL-12 levels, while the L, LP, BSP, and BS groups showed higher levels compared to the positive control but were significantly lower (**P* *< 0.0001) than the ALB group (Fig 3A and 3B).

The highest IFN-γ levels were observed at 5 dpi in the LP group, indicating an early strong Th1 response. This level decreased over time but remained significantly higher than that in the positive control group at 19 dpi. In the BSP group, the IFN-γ level at 5 dpi was significantly lower than in the LP and ALB groups but was still significantly higher (*P* < 0.0001) than in the positive control group. This level increased over time, reaching a significantly higher value at 19 dpi, although it was still lower than those in the LP and ALB groups. The IFN-γ level in the positive infected control group increased over time, peaking at 19 dpi and surpassing the levels in the positive control group and other groups. The L and BS groups showed significantly (*P* < 0.0001) lower levels of IFN-γ throughout the experiment compared to the positive infected control group and other groups (Fig 3C and 3D).

IFN-γ levels in the positive infected control group were significantly higher than in the positive control group and other groups at 19 dpi.

IL 17 levels at 5 dpi were significantly higher in the ALB group than in the positive control and other treatment groups. The LP group showed no statistically significant difference in comparison with the ALB group at 5 dpi. However, at 19 dpi the level of IL 17 was significantly (*P* < 0.0001) lower than that in the ALB group. The L and BS groups had the lowest IL 17 levels among all groups however this level is higher over time at 19 dpi but still significantly (*P* < 0.0001) lower than that in the ALB group (Fig 3E and 3F).

#### 3.4.2. Serum anti-*Trichinella* IgG response.

The ELISA results showed that the serum anti-*Trichinella* IgG levels in the LP and BSP groups at 19 days post-infection were significantly higher than at 5 days post-infection (**P* *<* *0.05). However, the IgG levels in the LP group were significantly higher (*P* <* *0.05) than those in the other groups, suggesting that *L. brevis* PQ214320 triggered a stronger IgG immune response than *B. subtilis* PQ198038, despite both being administered orally before and after infection.

**Fig 4:**
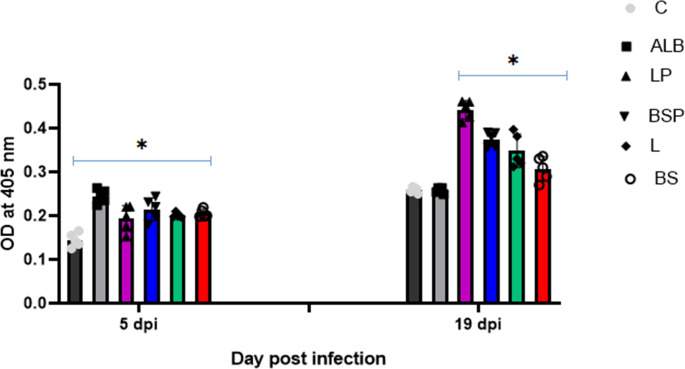
Detection of anti-*Trichinella* IgG in mice tested groups at 5 dpi and 19 dpi by ELISA. All serum samples were tested in triplicate. The data are presented as the mean OD values ± standard deviation (SD) of 5 mice per group of anti-*Trichinella* IgG level. The anti-*Trichinella* IgG level was detected using an indirect ELISA. One-way ANOVA, post hoc Tukey’s test for multiple comparisons was used. * Mean significance difference (*P < *0.05) in comparison with control infected non-treated group **(C)**. (ns) indicate non-significance difference in comparison with the C group.

Moreover, mice orally treated with *B. subtilis* PQ198038 and *L. brevis* PQ214320 only after infection showed high IgG levels at 19 dpi compared to those at 5 dpi, indicating an adaptive immune response over time. However, there were no significant differences in IgG responses at 19 days post-infection. However, their IgG levels were significantly lower than those of the LP and BSP groups (Fig 4).

#### 3.5.1. Histopathological and histochemical findings in mice small intestine and diaphragm muscles.

**Fig 5:**
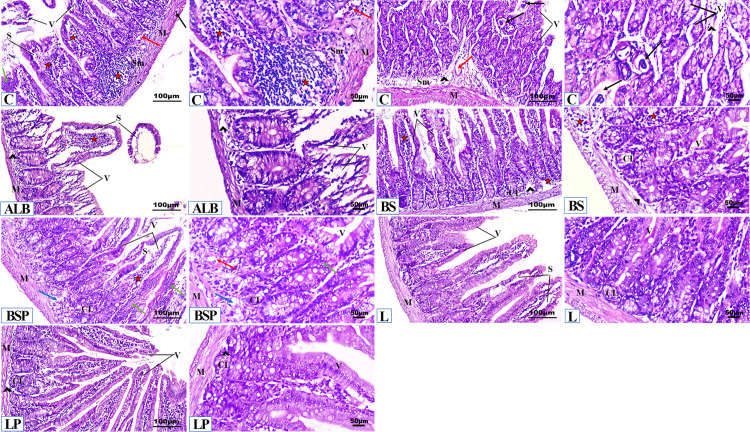
Representative histopathological photomicrographs of small intestine in the different experimental mice groups (n = 5). C: Positive *Trichinella* untreated control mice showing thickening and flat of the villi **(V)**, degeneration, atrophy, necrosis with sloughing of the upper tips of the villi **(S)**, multiple *T. spiralis* larvae (arrow) in the villus cryptus junctions and at muscularis (M) associated with severe infiltration of inflammatory cells in submucosa (Sm) and lamina propria (star), hyperplasia of goblet cells (green arrow), dilatation of the blood capillary (red arrow), and edema in different intestinal layers (arrowhead) (H&E, low × 200 and high ×400 Magnifications). ALB: Infected mice treated with Albendazole, ALB group displaying sub-epithelial cell edema (arrowhead), atrophy, and sloughing (S) of the upper tips of some villi (V) with infiltration of inflammatory cells (star), muscular layer **(M)** (H&E, low × 200 and high ×400 Magnifications). BS: Infected mice post-treated with *B. subtilis* PQ198038, BS group exhibiting degeneration of the villi (V) with infiltration of inflammatory cells (star), and sub-mucosal edema (arrowhead) in-between crypts of Lieberkühn (CL), muscular layer **(M)** (H&E, low × 200 and high ×400 Magnifications). BSP: Infected mice pre-treated with *B. subtilis* PQ198038, before infection and continued after infection, BSP group displaying a sloughing (S) of the upper tips of some villi (V) with infiltration of inflammatory cells (star), degenerated crypts of Lieberkühn (blue arrow), hyperplasia of goblet cells (green arrow), and dilatation of the blood capillary (red arrow), muscular layer **(M)** (H&E, low × 200 and high ×400 Magnifications). L: Infected mice post-treated with *L. brevis* PQ214320, L group showing an improvement of villi (V) with a slight sloughing **(S)**, crypts of Lieberkühn (CL), muscular layer **(M)** (H&E, low × 200 and high ×400 Magnifications). LP: Infected mice pre-treated with *L. brevis* PQ214320, before infection and continued after infection, LP group exhibiting a noticeable improvement with slight signs of edema (arrowhead), crypts of Lieberkühn (CL), muscular layer **(M)** (H&E, low × 200 and high ×400 Magnifications).

**Fig 6:**
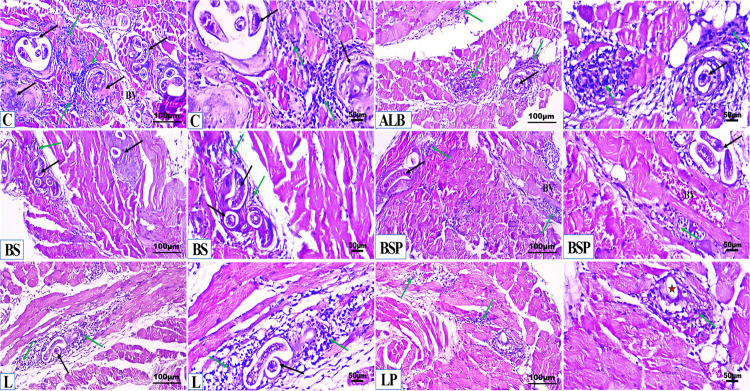
Representative histopathological photomicrographs of diaphragm muscle in the different experimental mice groups (n = 5). C: Positive *Trichinella* untreated control mice showing multiple *T. spiralis* larvae (black arrow) associated with a severe inflammatory cell infiltrate (green arrow), and congestion of blood vessels (BV) (H&E, low × 200 and high ×400 Magnifications). ALB: Infected mice treated with Albendazole, ALB group displaying damaged *T. spiralis* larvae (black arrow) associated with moderate infiltration of inflammatory cells (green arrow) (H&E, low × 200 and high ×400 Magnifications). BS: Infected mice post-treated with *B. subtilis* PQ198038, BS group exhibiting some *T. spiralis* larvae (black arrow) associated with a mild inflammatory cell aggregation (green arrow) (H&E, low × 200 and high ×400 Magnifications). BSP: Infected mice pre-treated with *B. subtilis* PQ198038, before infection and continued after infection, BSP group displaying a small number of *T. spiralis* larvae (black arrow) associated with a mild inflammatory cellular infiltration (green arrow), and congested blood vessels (BV) (H&E, low × 200 and high ×400 Magnifications). L: Infected mice post-treated with *L. brevis* PQ214320, 19 days after infection, L group showing a small number of *T. spiralis* larvae (black arrow) associated with a mild aggregation of inflammatory cells (green arrow) (H&E, low × 200 and high ×400 Magnifications). LP: Infected mice pre-treated with *L. brevis* PQ214320, before infection and continued to 19 days after infection, LP group exhibiting a noticeable improvement without any larvae (Star), with minimal aggregation of inflammatory cells (arrow) (H&E, low × 200 and high ×400 Magnifications).

Microscopic examination of the small intestine at 5 dpi (Fig 5) and diaphragm muscle at 19 dpi (Fig 6) sections of *Trichinella* infected untreated mice revealed severe disorganization of tissue morphology, inflammatory cell infiltration, degeneration, necrosis, and moderate edema with a marked existence of multiple *T. spiralis* larvae. Infected mice treated with albendazole demonstrated minimal protective efficacy with a moderate infiltration of inflammatory cells. The BS and BSP groups exhibited moderate protective effects, with approximately normal histological characteristics of the tissues and minimal to mild infiltration of inflammatory cells. The L and LP groups demonstrated noticeable improvements in the histological organization of intestinal and muscular tissues with slight signs of degeneration.

**Fig 7:**
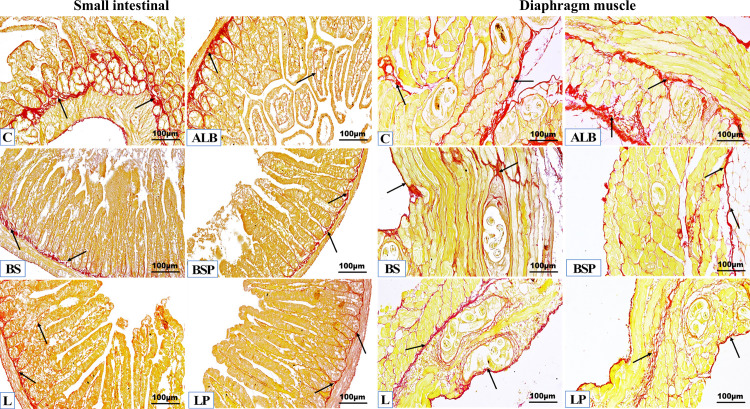
Representative photomicrographs of intestinal and diaphragm muscular sections stained by Picrosirius red distinguishing the organization of collagen fibers (red color, arrows), cytoplasm and muscle fibers appeared as a yellow color in the different experimental mice groups (n = 5). C: Positive *Trichinella* untreated control mice, ALB: Infected mice treated with Albendazole, BS: Infected mice post-treated with *B. subtilis* PQ198038, BSP: Infected mice pre-treated with *B. subtilis* PQ198038 before infection and after infection, L: Infected mice post-treated with *L. brevis* PQ214320, LP: Infected mice pre-treated with *L. brevis* PQ214320 before infection and continued after infection, (SR × 200).

**Fig 8:**
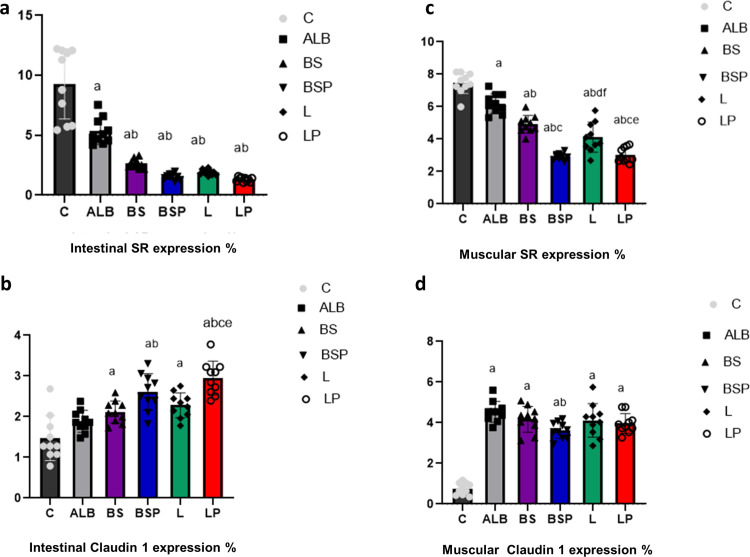
All the intestinal (a,b) and muscular (c,d) Picrosirius red and Claudin 1 expression values are expressed as the mean ± SD (n = 5, 10 random fields per each one). C: Positive *Trichinella* untreated control mice, ALB: Infected mice treated with albendazole, BS: Infected mice post-treated with *B. subtilis* PQ198038, BSP: Infected mice pre-treated with *B. subtilis* PQ198038 before of infection and continued after infection, L: Infected mice post-treated with *L. brevis* PQ214320, LP: Infected mice pre-treated with *L. brevis* PQ214320 before of infection and continued after infection. The significant (*P < *0.01) differences are (a) compared to the C group, (b) against the ALB group, (c) compared with the BS group, (d) compared with the BSP group, and (e) compared with L group. The significant (*P* < 0.05) difference is (^f^) compared to the C group. The remaining groups representing insignificant difference relative to each other’s.

Quantitative analysis of the intestinal and diaphragm muscular tissue sections (Figs 7 and 8) revealed significantly *P* < 0.01 reduced levels of fibrosis in all treated groups relative to the infected untreated group, all *B. subtilis* PQ198038 and *L. brevis* PQ214320 groups recorded a significant decrease *P* < 0.01 compared to infected mice treated with Albendazole, and insignificant changes were recorded between all *B. subtilis* PQ198038 and *L. brevis* PQ214320 treatments in intestinal tissue, while significant decreases were recorded in the muscular sections of BSP group (*P* < 0.01), or L group (*P* < 0.05), or LP group (*P* < 0.01) revealed to BS group, as well as, an insignificant change was observed between BSP group against LP group. *Trichinella*-infected untreated mice exhibited the maximum fibrosis in the intestine (9.29 ± 2.93) and muscle (7.45 ± 0.65). Infected mice treated with Albendazole recorded moderate to strong significant fibrosis in tissue sections of the intestine (5.33 ± 1.19) and muscle (6.19 ± 0.61). BS group demonstrated mild to moderate fibrosis in tissue sections of the intestine (2.63 ± 0.38) and muscle (4.92 ± 0.54). The BSP group displayed minimal to mild fibrosis in tissue sections of the intestine (1.56 ± 0.24) and muscle (2.92 ± 0.19). L group showed mild to moderate fibrosis in tissue sections of the intestine (1.91 ± 0.21) and muscle (4.11 ± 0.92). LP group exhibited minimal to mild fibrosis in tissue sections of the intestine (1.31 ± 0.17) and muscle (3.01 ± 0.45).

#### 3.5.2. Immunohistochemical examinations.

**Fig 9:**
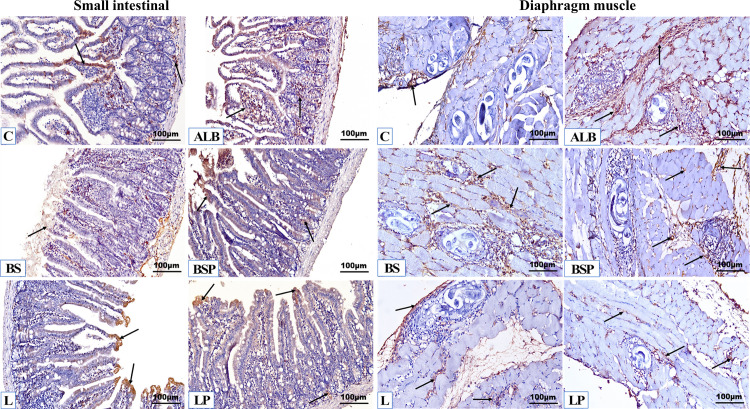
Representative photomicrographs of the immunohistochemical membranous Claudin-1 reactions (brown color, arrows) lining the total borders in the intestinal and muscular tissues of the different experimental mice groups (n = 5). C: Positive *Trichinella* untreated control mice, ALB: Infected mice treated with Albendazole, BS: Infected mice post-treated with *B. subtilis* PQ198038, BSP: Infected mice pre-treated with *B. subtilis* PQ198038 before infection and continued after infection, L: Infected mice post-treated with *L. brevis* PQ214320, LP: Infected mice pre-treated with *L. brevis* PQ214320 before 7 days of infection and continued to 5 days after infection, (IHC, × 200).

Quantitative analysis of brown Claudin-1 positive expression of the intestinal and diaphragm muscular tissue sections (Figs 8 and 9) revealed a significant increase *P* < 0.01 in all treatments compared to infected untreated mice, a significant increase *P* < 0.01 in the BSP group compared to infected mice treated with ALB, LP group recorded a significant increase *P* < 0.01 in the intestinal tissue compared to the BS and L groups, while the remaining groups showed insignificant changes compared to each other. The infected untreated mice exhibited few positive immunohistochemical reactions in tissue sections of the intestine (1.47 ± 0.30) and muscle (0.75 ± 0.29). Revealed to the positive control group, it became clear that apparent restorations of Claudin-1 were recorded in infected mice treated with ALB, BS, BSP, L, and LP in tissue sections of the intestine (1.88 ± 0.07, 2.11 ± 0.07, 2.61 ± 0.20, 2.28 ± 0.09, and 2.94 ± 0.17; respectively) and muscle (4.54 ± 0.51, 4.17 ± 0.63, 3.63 ± 0.40, 4.11 ± 0.82, and 3.97 ± 0.48; respectively).

## 4. Discussion

Probiotics must exhibit good safety profile for human use, including non-pathogenicity and resistance to commonly used antibiotics. In the current study, *L. brevis* PQ214320 and *B. subtilis* PQ198038 showed safety profile as the strains are not pathogenic and they are tolerant of a broad spectrum of antibiotics. Moreover, Both strains inhibited the growth of *S. aureus*, *E. coli*, *K. pneumoniae* and *P. aeruginosa*

*L. brevis* PQ214320 and *B. subtilis* PQ198038 significantly reduced both adult worm and muscle larval counts of *T. spiralis* in experimentally infected mice. Notably, *L. brevis* exhibited better efficacy compared to *B. subtilis* across all treatment groups. The LP group (treated with *L. brevis* before and after infection) showed a significant decrease in adult worm burden and larval count compared to the positive control group (P < 0.0001). The BSP group (pre- and post-infection *B. subtilis*) also demonstrated reductions, though to a lesser extent. When treatment was administered post-infection only (L and BS groups), larval burdens were higher, this results confirm the benefit of prophylactic use.

These findings are consistent with previous reports on the antiparasitic potential of probiotics such as *L. casei* and *L. plantarum* [13,14,48], and highlight the strain-specific nature of probiotic effects [49]. The observed efficacy may result from multiple mechanisms: secretion of antiparasitic metabolites (e.g., lactic acid, hydrogen peroxide, and bacteriocins), interference with parasite reproduction and development, increased intestinal motility, and immune modulation [50,51].

This study provides critical insights into how different treatments modulate the immune responses against *Trichinella* infection in experimentally infected mice. Specifically, the analysis of IL 12, IFN-γ, and IL 17 cytokine levels, in addition to anti-*Trichinella* IgG responses at different intervals of infection, a comparative perspective on the immunomodulatory effect of *L. brevis*, *B. subtilis*, and albendazole. At 5 dpi, the LP group exhibited the highest IL 12 levels, indicating of a robust early immune response. The ALB, and LP groups also showed elevated IL 12 levels compared to the positive control group, with the L and BS groups showing lower levels. At 19 dpi, the ALB group displayed significantly higher IL-12 levels than the other groups. IL 12 is crucial for initiating the Th1 response, which aids in intracellular parasite control. Previous studies have shown that probiotics, particularly *Lactobacillus* spp., enhance IL 12 production and, promote early immune activation against parasites [26]. Similar findings with *L. casei* confirmed that certain probiotics can stimulate IL 12 and IFN-γ production, offering protection against *T. spiralis* [31]. However, sustained IL 12 elevation with albendazole treatment suggests prolonged inflammation, which may align with studies indicating that drug treatments can trigger an extended immune response without controlled modulation [14]. The LP group also demonstrated the highest IFN-γ levels at 5 dpi, which then declined over time but remained higher than the positive control group at 19 dpi. In contrast, the BSP group’s IFN-γ levels peaked later, reaching a higher level at 19 dpi but still lower than LP and ALB. IFN-γ is a central component of Th1 immunity, essential for parasite control through macrophage activation and parasite clearance. Similar studies with recombinant *L. plantarum* in mice showed that IFN-γ production facilitated suppression of *T. spiralis* [52]. The delayed IFN-γ response in the BSP group may indicate a slower immune activation with *B. subtilis*, possibly affecting its effectiveness relative to *L. brevis*. Moreover, Mazziotta et al. [53], illustrated that *L. casei* and *L. plantarum* boost cell-mediated immunity via Th1 responses by activating dendritic cells (DCs) and macrophages, which release IL-12, subsequently inducing IFN-γ production in T-cells. This mechanism may explain why *L. brevis* was particularly effective in inducing these cytokines early in the infection, which is beneficial for controlling *T. spiralis*.

The better performance of *L. brevis* PQ214320 than *B. subtilis* PQ198038 in reducing both adult worm and muscle larval burdens may be attributed to differences in immune modulation mechanisms. *L. brevis* induced earlier and stronger production of IL 12 and IFN-γ, which are crucial for initiating Th1 responses and activating macrophages, leading to enhanced parasite clearance. Additionally, *L. brevis* may exhibit better colonization efficiency in the intestinal environment, allowing it to interact more effectively with gut-associated lymphoid tissues. Differences in metabolite production, such as higher levels of lactic acid, bacteriocins, or other bioactive molecules, may also contribute to its enhanced antiparasitic activity. Future studies should investigate these mechanisms in more depth to clarify how strain-specific traits influence therapeutic efficacy.

Although use of pre-treatment with probiotics prior to *Trichinella* exposure may appear to be not practical in clinical human cases due to the accidental nature of infection, the regular dietary use of probiotics particularly in endemic areas may enhance baseline mucosal immunity and reduce the severity of infection. Thus, promoting the routine intake of beneficial probiotic strains such as *L. brevis* through functional foods or supplements could serve as a supportive preventive strategy, especially for individuals in high-risk environments where consumption of undercooked meat is common. Further clinical studies are warranted to evaluate the timing, dosage, and strain specificity of probiotics for human applications.

In the current study, concerning the variable histological and histochemical assessments (H&E and Picrosirius red staining) examinations of intestinal and muscular sections from infected untreated mice, a massive invasion of *T. spiralis* encysted larvae, diffuse degenerative modifications with a heavy infiltrate of inflammatory cells, and severe fibrosis were observed. This tissue damage may be attributed to various mechanisms such as the oxidative stress accompanied by high production of inflammatory cell which produced an excessive amount of reactive oxygen species, as reported by Elgendy et al. [54]. Similar results were reported by Attia et al. [55] and Eid et al. [56], who discovered that muscle sections from *T. spiralis* infected untreated mice showed a focus on inflammatory cell infiltration around larvae and the infected muscle cells. Shalaby et al. [57] and Eissa et al. [58] reported similar results in *T. spiralis*-infected rats with different cellular responses. *Trichinella* infection induces marked intestinal and muscular inflammations [59–61]. Ding et al. [62] confirmed an increase in immune cell responses in the intestinal sections of *T. spiralis*-infected mice. Similarly, the development of cellular infiltration around the nurse cells of *T. spiralis* in experimental *Trichinella* infection in mice was detected [63]. This outcome may explain the diversity in the inflammatory infiltration degree between the present work and other studies.

Albendazole treatment improved the histological and histochemical results relative to the untreated infected group in the current study. Examination of intestinal and muscle sections from the ALB group revealed moderate infiltration of inflammatory cells and fibrosis. These results are consistent with the findings of Nassef et al. [64], Eid et al. [56], and Salama et al [65], who reported have previously stated that animals treated with ALB displayed a massive inflammatory cell infiltration in experimental *Trichinella* infection models. In contrast, Shalaby et al. [57] reported that rats treated with ALB exhibited only a few local infiltrations of inflammatory cells during the muscular phase. Similarly, Nada et al. [66] illustrated a mild inflammatory infiltrate around the larvae in muscular sections of ALB-treated mice. The ABZ-treated group exhibits a significant reduction in both intestinal and muscular inflammation during experimental *Trichinella* infection [61]. Conversely, Ibrahim et al. [67] recorded the least improvement in all pathological intestinal and muscular alterations caused by *T. spiralis* infections.

Regarding *B. subtilis* PQ198038 and *L. brevis* PQ214320 treatments, the current study outcomes recorded a significant improved degree of inflammation and reduction of fibrosis in the *Bacillus* and *Lactobacillus* treatment groups revealed to the infected untreated group. The anti-inflammatory and anti-fibrotic effect of *Bacillus* or *Lactobacillus* may be due to the release of a variety of growth factors upon the activation of the platelets, which act as anti-inflammatory agents by blocking monocyte chemotactic protein-1 production and reducing fibrosis by suppressing the production of collagen, as reported by El-Sharkawy et al. [68]. In recent decades, there has been much research and development on the use of probiotics to prevent and treat intestinal diseases. El Temsahy et al. [14] assessed the protective efficacy of Egyptian probiotic strains against pathological and immunological changes induced by intestinal *T. spiralis* infection. Bucková et al. [69] reported the effect of six selected probiotic strains on the intensity of trichinellosis. To our knowledge, no previous studies have investigated the histological effects of *B. subtilis* PQ198038 and *L. brevis* PQ214320 on *T. spiralis* infection. *Lactobacillus* has a strong anti-inflammatory effect on bowel inflammation [70]. Administration of the commercial probiotics *Linex* and ALB ameliorated the pathological changes and improved the immune response of the intestinal and muscular tissues of *T. spirals*-infected mice [71]. Finally, the histopathological assessment results in the current study were consistent with the parasitological and immunological assessment results, as the groups treated with *B. subtilis* PQ198038 and *L. brevis* PQ214320 demonstrated a significant improvement in inflammation and fibrosis, which are induced as side effects of *T. spirals* infection.

In the present study, apparent curative efficiency for tight junctions Claudin-1 expressions was recorded on intestinal and muscular sections of all treatment groups revealed to the infected untreated group. Similarly, Le et al. [72] reported that the epithelial barrier of the gastrointestinal canal with tight junctions such as Claudins acts as the first line of defense against injury, inflammation, infections, and pathogens. Similarly, Ahmed and Sheir [73] concluded that the elevation of Claudin-1 in the duodenum and colon tissue sections confirmed the ameliorative regulation of Hibiscus in an inflammatory bowel diseases (IBD) model. Xia et al. [74], and Ahmed and Sheir [73] reported similar decreases in expression of Claudin-1 in experimental IBD models that affected epithelial tight junctions. Furthermore, Zhu et al. [75] stated that downregulation of Claudin-1 participated in the pathogenesis of IBD are contributed to the elevation of intestinal permeability via NF-kB activation. Administration of *Lactobacillus* GG restores the epithelial barrier disruption caused by gliadin [76]. *L. reuteri* I5007 modulated the expression of tight junction proteins in a porcine jejunal epithelial cell line (IPEC-J2) following lipopolysaccharide (LPS) stimulation [77]. *Lactiplantibacillus plantarum* BSG201683 ameliorates LPS-induced intestinal inflammation and epithelial barrier damage caused by LPS [78]. *Bacillus pumilus* SMU5927 improves intestinal injury and significantly enhances intestinal barrier function [79]. *L. gasseri* JM1 alleviated ulcerative colitis in mice by ameliorating intestinal barrier disruption [80]. Qian et al. [81] confirmed that *L. gasseri* ATCC33323 ameliorated the intestinal mucosal barrier in the colitis model by regulating tight junction proteins like Claudin1 proteins.

## 5. Conclusion

The present study revealed that *Bacillus subtilis* PQ198038 and *Lactobacillus brevis* PQ214320 treatments had a modulatory efficiency against the pathogenesis effects induced by *T. spiralis* larvae on the intestinal and muscular phase of *Trichinella* infection in the experimentally infected mice. So, expand the therapeutic use of these strains alone potentially as a treatment in combination with the standard chemotherapeutic agents for other parasitic diseases, particularly those affecting the intestine or muscles. While further studies are needed, especially in clinical or larger animal models, the promising results of this study support the exploration of these strains as standalone treatments or as adjuncts to conventional antiparasitic therapies. *T. spiralis* is a foodborne zoonotic parasite of global concern Enhancing resistance to infection through routine probiotic intake especially in high-risk populations consuming undercooked meat may offer a low cost, accessible, and safe supplementary strategy. These findings may also guide future research toward developing probiotic based preventive or adjunct therapies in both veterinary and human medicine.

## Supporting Information

S1 Data Excel file containing raw and analyzed data related to immunological and parasitological and pathological outcomes in different experimental groups. The file includes the following sheets: Concentrations of IL 17 measured in serum samples across tested groups. Concentrations of IL 12 measured in serum samples across tested groups. Concentrations of IFN**-γ** measured in serum samples across tested groups. Anti-*Trichinella* IgG levels in mice tested groups. The intestinal and muscular SR expression values. The intestinal and muscular Claudin 1 expression values. Total worm counts observed in tested groups. Larval burden measured in muscle tissue.
(XLSX)
